# Modulation of cytokine production by carnitine

**DOI:** 10.1155/S0962935193000717

**Published:** 1993

**Authors:** Nicola M. Kouttab, Claudio De Simone

**Affiliations:** 1 Department of Pathology Roger Williams Medical Center and Brown University Providence Rhode Island USA; 2 Infectious Diseases Department of Internal Medicine University of L'Aquila L'Aquila Italy

## Abstract

The ability of carnitine congeners to modulate cytokine production by human peripheral blood mononuclear cells (PBMC) was investigated. Modulation of cytokine production by PBMC of young (30 years of age or younger) and old (70 years of age or older) normal donors was first compared. The PBMC were collected over Ficoll–Hypaque and incubated in the presence of various concentrations of acetyl L-carnitine for 24 h. Subsequently the supernatants were collected and examined for cytokine production. The presence of cytokines in tissue culture supernatants was examined by ELISA. The cytokines measured included IL-1α, IL-1β, IL-2, IL-4, IL-6, TNFα, GM–CSF, and IFNγ. The results showed that at 50 μg/ml of acetyl L-carnitine the most significant response was obtained for TNFα. In this regard four of five young donors responded, but only one of five old donors responded. More recently these studies were expanded to examine the ability of L-carnitine to modulate cytokine production at higher doses, 200 and 400 μg/ml, in young donors. The results of these studies showed that in addition to TNFα, significant production of IL-1β and IL-6 was observed. These preliminary studies provide evidence that carnitine may modulate immune functions through the production of selected cytokines.


Mediators of Inflammation 2, S25–S28 (1993)

### Modulation of cytokine production by carnitine

Nicola M. Kouttab[1,CA] and Claudio De Simone[2]

[1]Department of Pathology, Roger Williams Medical Center and Brown University, Providence, Rhode Island, USA;
[2]Infectious Diseases, Department of Internal Medicine, University of L'Aquila, L'Aquila, Italy

[CA]Corresponding Author

**THE ability of carnitine congeners to modulate cytokine production by human peripheral blood mononuclear cells (PBMC) was investigated. Modulation of cytokine production by PBMC of young (30 years of age or younger) and old (70 years of age or older) normal donors was first compared. The PBMC were collected over Ficoll–Hypaque and incubated in the presence of various concentrations of acetyl L-carnitine for 24 h. Subsequently the supernatants were collected and examined for cytokine production. The presence of cytokines in tissue culture supernatants was examined by ELISA. The cytokines measured included IL-1α, IL-1β, IL-2, IL-4, IL-6, TNFα, GM–CSF, and IFNγ. The results showed that at 50 μg/ml of acetyl L-carnitine the most significant response was obtained for TNFα. In this regard four of five young donors responded, but only one of five old donors responded. More recently these studies were expanded to examine the ability of L-carnitine to modulate cytokine production at higher doses, 200 and 400 μg/ml, in young donors. The results of these studies showed that in addition to TNFα, significant production of IL-1β and IL-6 was observed. These preliminary studies provide evidence that carnitine may modulate immune functions through the production of selected cytokines.**

**Key words:** Cytokines, Human peripheral blood cells, TNFα

### Introduction

Long-chain fatty acids are a major source for the production of energy in humans. This is particularly true for various organs including the liver, the heart, and the skeletal muscle system.[1–3] Energy is generated through β-oxidation of these fatty acids which are transported by carnitine across the mitochondrial membrane.[1–3] In addition to its role as a carrier for long-chain fatty acids, carnitine has been implicated in other disorders. It has been demonstrated that L-carnitine *O*-palmitoyltransferase deficiency is accompanied by hypoketoic hypoglycaemia and cardiomyopathy.[4] This observation may indicate a potential role for carnitine in cardiac disease.[5–7] Several reports have pointed to the role of carnitine in the immune system. De Simone and colleagues have noted that an increase in carnitine concentration in the serum can potentiate certain functions such as mixed lymphocyte reactions and chemotaxis,[8] as well as increase lymphocyte response to mitogens.[9] Other studies have also shown that treatment of carnitine depleted post operative patients with L-carnitine enhances lymphocyte mitogenesis.[10] Improvement of immune functions during stress or ageing upon treatment with carnitine has also been well documented.[11,12] Recent studies also showed that AIDS patients demonstrate a deficiency in L-carnitine which may compromise their energy supplies.[13] These observations provide evidence that carnitine may be an important factor in the normal function of the immune system.

In the present studies the effect of carnitine congeners on the modulation of cytokine production by human peripheral blood mononuclear cells (PBMC) was examined. The results provide evidence that acetyl L-carnitine, and L-carnitine are capable of inducing the production of selected cytokines. Among the cytokines produced, IL-1β and IL-6 were produced. However, it was also observed that significant concentrations of tumour necrosis factor alpha (TNFα) were produced. The measurement of TNFα by ELISA was confirmed by demonstrating the presence of messenger RNA using the polymerase chain reaction (PCR) technique.

### Materials and Methods

Both acetyl L-carnitine and L-carnitine were a generous gift from Sigma Tau, Pomezia, Rome, Italy.

*Peripheral blood cells:* Peripheral blood was obtained from normal donors by venous puncture, using

© 1993 Rapid Communications of Oxford Ltd heparinized vacutainer tubes (Becton–Dickinson, San Jose, CA). Alternatively, when large numbers of cells were needed, blood was obtained from the Rhode Island Blood Center. The blood was centrifuged over Ficoll–Hypaque gradients as has been described previously.[14,15] The buffy coat cells at the interface of the gradient were collected and washed to remove the Ficoll. The cells were then resuspended in RPMI medium supplemented with glutamine (200 mM) and penicillin/streptomycin (100 units/ml). These cells were then used for the experiments as needed.

*Production of cytokines:* For cytokine production, PBMC in supplemented RPMI medium were adjusted to 1 × $10^6$ cells/ml in supplemented RPMI medium containing 2% heat inactivated foetal calf serum (FCS).[16] Acetyl L-carnitine or L-carnitine were added at various concentrations to cell cultures. Cultures were incubated at 37°C and 5% $CO_2$ in a humidified atmosphere for 24 h. Following incubation, the supernatants were harvested and filtered through a 0.2 μm filter (Gelman). The supernatants were divided into aliquots and frozen at −70°C until needed. Control cultures included cells incubated without carnitine, or cultures incubated with 5 μg/ml concancavalin A (Sigma).

*Cytokine measurement:* The presence of cytokines in supernatant cultures was measured by using enzyme linked immunosorbent assays (ELISA). The kits for these assays were purchased from Genzyme (Boston, MA) or R&D systems (Minneapolis, MN). All assays were performed in accordance with the manufacturer's instructions. The washing steps were done using a plate washer (ICN, Costa Mesa, CA) and the completed assays were read on an MS2 plate reader (ICN) to which an Okidata printer was attached. The concentrations of the cytokines in the culture supernatants were determined by reference to a set of standards included in the ELISA kit.

### Results and Discussion

The results of experiments comparing cytokine production by acetyl-L-carnitine in young and old normal individuals are summarized in Table 1. As can be seen, at the maximum dose of 50 μg/ml of acetyl L-carnitine used in these experiments, there was significant production of TNFα. The results show that significant concentrations of TNFα were produced by cells from four of five young donors, but only one old donor showed significant response. No measurable amounts of other cytokines were observed in these initial experiments. A graphic representation of this TNFα response is presented in Fig. 1. No or minimal TNFα (<10 pg/ml) was produced by cells cultured

**Table 1.** Modulation of cytokine production by acetyl L-carnitine

| Cytokine | Young donors < 30 y | Old donors > 70 y |
|---|---|---|
| GM-CSF | 0/5 | 0/5 |
| IFN-γ | 0/5 | 0/5 |
| TNF-α | 4/5 | 1/5 |
| IL-1 | 0/5 | 0/5 |
| IL-2 | 1/5 | 1/5 |
| IL-4 | 0/5 | 0/5 |
| IL-6 | 1/5 | 0/5 |

Human peripheral blood mononuclear cells (PBMC) were incubated with or without acetyl L-carnitine at various doses ranging from 0.01 to 50 μg/ml, for 24 h. The supernatants were collected and examined by ELISA for the presence of cytokines. The results above are from cultures incubated with 50 μg/ml acetyl L-carnitine

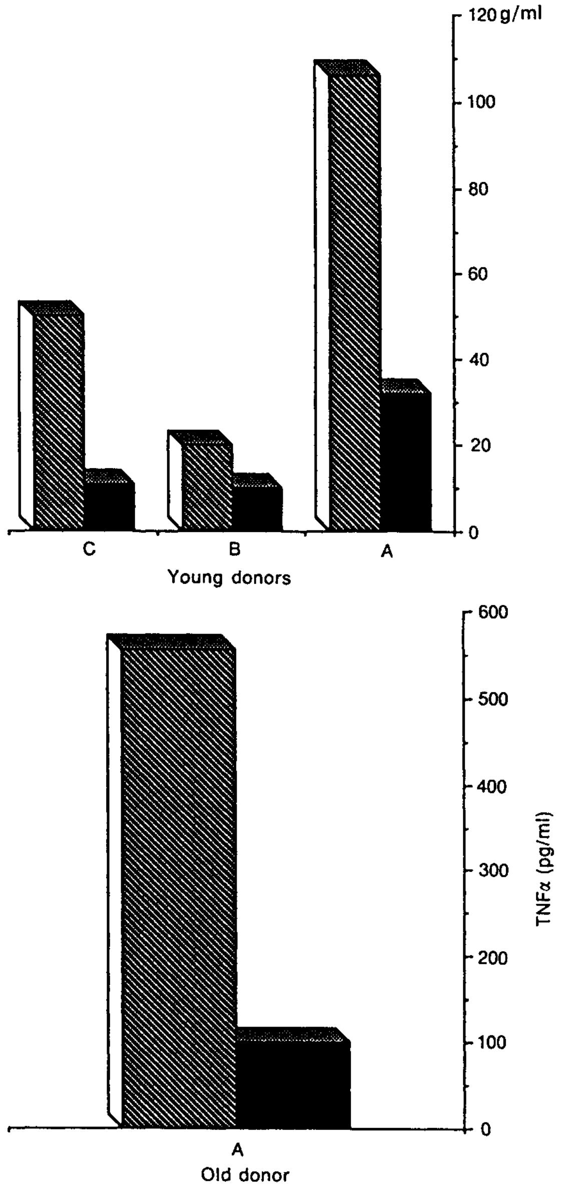

FIG. 1. Induction of TNFα by acetyl L-carnitine, in peripheral blood cells obtained from young (<30 years of age) and old (>70 years of age) normal human donors. The maximum induction in this experiment post 24 h of incubation was about four times that of cells incubated without acetyl L-carnitine. ■ cells alone; ▨ cells + acetyl-L-carnitine. Cells incubated with concanavalin A were used as a positive control producing approximately 700 pg/ml. Cytokine measurement was done by ELISA.

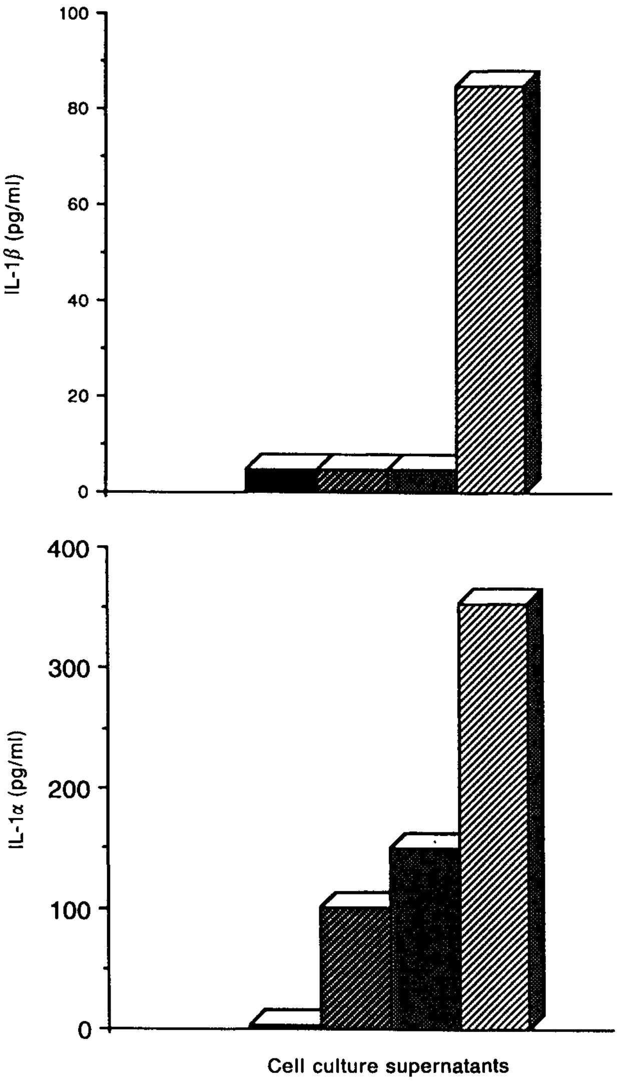

FIG. 2. Induction of IL-1β, but not IL-1α in peripheral blood of normal human cells, following incubation for 24 h in the presence of L-carnitine. Cells incubated with concanavalin A were used as a positive control. IL-1β levels increased from 4 pg/ml for cells without L-carnitine to 45 pg/ml and 100 pg/ml when cells were incubated with 200 and 400 μg/ml of L-carnitine, respectively. Cytokine measurement was done by ELISA. ■, cells alone; ▨, cells+L-carnitine (200 μg/ml); ■, cells+L-carnitine (400 μg/ml); ▨, cells+Con A.

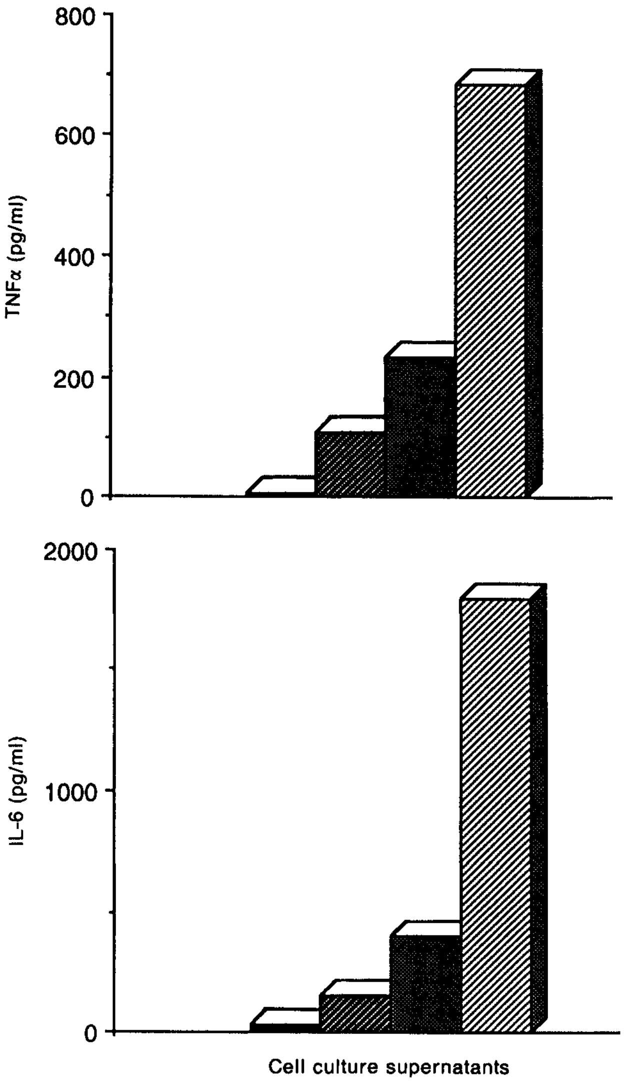

FIG. 3. Induction of IL-6 by normal human peripheral blood cells incubated with L-carnitine. IL-6 levels rose from virtually 0 pg/ml for cells without L-carnitine to 150 and 400 pg/ml in the presence of 200 and 400 μg/ml L-carnitine, when incubated for 24 h. Similar results were obtained for TNFα with these concentrations of L-carnitine. TNFα levels increased from virtually 0 pg/ml to 110 and 234 pg/ml, respectively. ■, cells alone; ▨, cells+L-carnitine (200 μg/ml); ■ cells+L-carnitine (400 μg/ml); ▨, cells+Con A.

in the absence of acetyl L-carnitine, which when added at 50 μg/ml induced significant concentrations of the cytokine, in some donors being as high as five times that of unstimulated cells. These results provide evidence that acetyl L-carnitine can modulate the production of cytokines by human PBMC. It appears however, that this process of cytokine production is more pronounced in cells of young donors than in cells obtained from old donors. This observation needs to be confirmed by

**Table 2.** Modulation of cytokine production by L-carnitine

| Supernatant | TNFα | IL-6 | IL-1β | IL-1α |
|---|---|---|---|---|
| Cells alone | 5 pg/ml | <10 pg/ml | 4 pg/ml | <5 pg/ml |
| Cells + L-car 200 | 110 pg/ml | 150 pg/ml | 101 pg/ml | <5 pg/ml |
| Cells + L-car 400 | 234 pg/ml | 400 pg/ml | 151 pg/ml | <5 pg/ml |
| Cells + Con A | 685 pg/ml | 1800 pg/ml | 354 pg/ml | 100 pg/ml |

Human peripheral blood mononuclear cells were incubated with or without L-carnitine (at 200 and 400 μg/ml) for 24 h. The supernatants were harvested and examined by ELISA for the presence of cytokines. Cells with concanavalin A (5 μg/ml) were included as a positive control using larger numbers of donors, before a conclusion can be made.

The above experiments were extended in young donors using L-carnitine instead of acetyl L-carnitine. In addition, the concentrations of L-carnitine used were higher than those used for acetyl L-carnitine, being 200 and 400 μg/ml. Figure 2 shows the results for one of at least three separate experiments for IL-1α, and IL-1β, all of which gave similar results. It is clear from the data that IL-1β but not IL-1α is produced in the presence of L-carnitine. The data for these experiments are summarized in Table 2. Similar results were obtained for IL-6 and for TNFα which can be seen in Fig. 3. Of importance in these experiments is that the concentrations of the cytokines increased in response to increased concentrations of L-carnitine (Table 2). Of the cytokines measured to date it appears that the induction of TNFα is observed at concentrations of carnitine which are not inductive for other cytokines. Thus at 50 μg/ml of acetyl L-carnitine there was strong induction of TNFα but not other cytokines. It should be mentioned, however, that oral administration of acetyl L-carnitine did not result in significant modifications of TNFα levels.[17] In the present study, the small number of donors has to be taken into consideration before conclusions can be drawn. Nevertheless, one also has to consider that this selection for TNFα production may be associated with some unique features of the ability of carnitine to modulate the immune response.

### References

1. Bieber LL, Farrell S. Carnitine octanoyltransferase of mouse liver peroxisomes: properties and effect of hypolipidemic drugs. *Arch Biochem Biophys* 1983; **222**: 123–132.
2. Demaugre F, Bonnefont J-P, Colonna M, Cepanec C, Leroux J-P, Saudubray J-M. Infantile form of carnitine palmitoyltransferase II deficiency with hepatomuscular symptoms and sudden death: physiopathological approach to carnitine palmitoyltransferase II deficiencies. *J Clin Invest* 1992; **87**: 859–864.
3. Finocchiaro G, Taroni F, Rochi M, *et al.* cDNA cloning, sequence analysis, and chromosomal localization of the gene for human carnitine palmitoyltransferase. *Proc Natl Acad Sci USA* 1991; **88**: 661–665.
4. Taroni F, Verderio E, Fiorucci S, *et al.* Molecular characterization of inherited carnitine palmitoyltransferase II deficiency. *Proc Natl Acad Sci USA* 1992; **89**: 8429–8433.
5. Broderick TL, Quinney HA, Lopaschuk GD. Carnitine stimulation of glucose oxidation in the fatty acid perfused isolated working rat heart. *J Biol Chem* 1992; **267**: 3758–3763.
6. Pepine CJ. The therapeutic potential of carnitine in cardiovascular disorders. *Clin Ther* 1991; **13**: 2–21.
7. Siliprandi N, Di Lisa F, Menabio R. Clinical use of carnitine. Past, present, and future. *Adv Exp Med Biol* 1990; **272**: 175–181.
8. De Simone C, Delogu G, Fagioli A, *et al.* Lipids and the immune system are influenced by L-carnitine. A study in elderly subjects with cardiovascular diseases. *Int J Immunotherapy* 1985; **1**: 267–271.
9. De Simone C, Ferrari M, Lozzi A, Meli D, Ricca A, Sorice F. Vitamins and immunity: II Influence of L-carnitine on the immune system. *Acta Vitaminologica Et Enzymologica* 1982; **4**: 135–140.
10. Carlsson M, Sundqvist MP. L-Carnitine enhances lymphocyte mitogenesis in depleted traumatised and infected patients. *Clin Nutr* 1987; **6**: 39–44.
11. Angelucci L, Ramacci MT, Hypothalamo–pituitary–adrenocortical function in aging: effects of acetyl L-carnitine. In: De Simone C, Martelli EA, eds. *Stress, immunity and ageing. A role for acetyl L-carnitine.* Amsterdam: Elsevier Science Publishers, 1989; 97–107.
12. Monti D, Cossarizza A, Troiana L, Arrigoni-Martelli E, Franceschi C. Immunomodulatory properties of L-acetylcarnitine on lymphocytes from young and old humans. In: De Simone C, Martelli EA, eds. *Stress, immunity and ageing. A role for acetyl L-carnitine.* Amsterdam: Elsevier Science Publishers, 1989; 83–96.
13. De Simone C, Tzantzoglou S, Jirrillo E, Marzo A, Vullo V, Martelli EA. L-Carnitine deficiency in AIDS patients. *Curr Sci* 1992; **6**: 203–205.
14. Kouttab NM, Pathak S, Berger A, Sahasrabbudhe CG, Maizel AL. Establishment of stable human T–T hybridomas. *J Immunol Methods* 1985; **77**: 165–172.
15. Mokotoff M, Zhao M, Roth SM, Shelley JA, Slavoski JN, Kouttab NM. Thymosin-like peptides as potential immunostimulants. Synthesis via the polymeric reagent method. *J Med Chem* 1990; **33**: 354–360.
16. Kouttab NM, Goldstein A, Lu M, Lu L, Campbell B, Maizel AL. Production of human B and T cell growth factors is enhanced by thymic hormones. *Immunopharmacology* 1988; **16**: 97–105.
17. Jirillo E, Altamura M, Munno I, *et al.* Effects of acetyl L-carnitine oral administration on lymphocyte antibacterial activity and TYYNF alpha levels in patients with active pulmonary tuberculosis. A randomized double blind versus placebo study. *Immunopharmacol Immunotoxicol* 1991; **13**: 135–146.

ACKNOWLEDGEMENTS. The authors would like to thank Marya Koza for excellent technical assistance. Carnitine was a generous gift from Tau, Pomezia, Rome, Italy.
